# Multi-Omics Analysis of Diabetic Nephropathy Reveals Potential New Mechanisms and Drug Targets

**DOI:** 10.3389/fgene.2020.616435

**Published:** 2020-12-11

**Authors:** Qian Sha, Jinxiu Lyu, Meng Zhao, Haijuan Li, Mengzhe Guo, Qiang Sun

**Affiliations:** ^1^Department of Pharmacy, The Affiliated Hospital of Xuzhou Medical University, Xuzhou, China; ^2^Jiangsu Key Laboratory of New Drug Research and Clinical Pharmacy, Xuzhou Medical University, Xuzhou, China

**Keywords:** DN, multi-omics, fatty acid metabolism, linoleic acid, LC-MS/MS

## Abstract

Diabetic nephropathy (DN) is one of the most common diabetic complications, which is the major course of end-stage renal disease (ESRD). However, the systematical molecular characterizations during DN pathogenesis and progression has not been not well understood. To identify the fundamental mediators of the pathogenesis and progression of DN. we performed a combination RNASeq, proteomics, and metabolomics analyses of both patients’ derived kidney biopsy samples and kidneys from *in vivo* DN model. As a result, molecular changes of DN contain extracellular matrix accumulation, abnormal activated inflamed microenvironment, and metabolism disorders, bringing about glomerular sclerosis and tubular interstitial fibrosis. Specificity, Further integration analyses have identified that the linoleic acid metabolism and fatty-acids β-oxidation are significantly inhibited during DN pathogenesis and progression, the transporter protein ABCD3, the fatty acyl-CoA activated enzymes ACOX1, ACOX2, and ACOX3, and some corresponding metabolites such as 13′-HODE, stearidonic acid, docosahexaenoic acid, (±)10(11)-EpDPA were also significantly reduced. Our study thus provides potential molecular mechanisms for DN progression and suggests that targeting the key enzymes or supplying some lipids may be a promising avenue in the treatment of DN, especially advanced-stage DN.

## Introduction

Diabetic nephropathy (DN) is one of the worst diabetic complications, which develops in over 40% of type 2 diabetic (T2D) patients (Thomas et al., 2015). It seriously weakens the kidney function, sequentially leading to end-stage renal disease (ESRD) (Eckardt et al., 2013; Fineberg et al., 2013). The pathologic changes of DN contain renal hypertrophy and extracellular matrix accumulation, bringing about glomerular sclerosis and tubular interstitial fibrosis (Kato and Natarajan, 2019; Cole and Florez, 2020). The diagnosis of DN and its severity is currently based on clinical features such as glomerular filtration rate (GFR), proteinuria, and albuminuria, as well as histological changes observed in the kidney biopsy samples (MacIsaac and Ekinci, 2019; Sinha et al., 2019). Relevantly, proteinuria is the authoritative indicator for evaluating renal failure (Ronco and Debiec, 2020). At the early stage of DN (microalbuminuria), the renal injury can be alleviated by clinical treatment including the control of blood glucose and pressure according to previous reports. However, there existing no effective treatments for patients with advanced-stage DN (Muskiet et al., 2019). Therefore, new biomarkers must be discovered for helping the clinical evaluation of diagnosis and prognosis to delay the progression of DN. So far, the pathogenesis of DN has not yet been clearly elucidated, which is difficult for doctors to make appropriate treatments for DN patients. Thus, analyzing of DN at different levels would be helpful to explore the mechanisms of DN pathogenesis and progression.

The improvements of next-generation sequencing and Liquid chromatography-mass spectrometry (LC-MS)have accelerated the studies at the multi-omics level, including genomics, transcriptomics, epigenomics, and proteomics, which largely promote the studies of biomarker identifications and mechanism investigations of complex diseases including different types of cancer, T2D, kidney diseases, and so on (Yang et al., 2015; Huang et al., 2017). The past decade has witnessed the new era of identifying new biomarkers and drug targets of different kidney diseases including DN using the NGS and LC-MS techniques (Bontha et al., 2017; Eddy et al., 2020). Notably, the multi-omics data such as proteome, transcriptome, genome, and metabolome provide numerous resources for building new early diagnosis models and developing effective therapeutic strategies for DN patients and eventually improve their prognosis. These data have also aided the molecular mechanism investigations of DN pathogenesis and progression, and lead to building of several models for evaluating the response of certain treatments in DN patients (Filla and Edwards, 2016; Van et al., 2017; Fan et al., 2019). Therefore, the researchers would gain a comprehensive view of DN by integrating multi-omics data such as proteome, transcriptome and so on, which will help develop new drugs and provide personalized therapies for individual DN patients.

In this study, we systematically analyze the landscape molecular alterations by integrating proteome, transcriptome, and metabolome during DN pathogenesis and progression. The results revealed that there is existing extracellular matrix accumulation, abnormal activated inflamed microenvironment, and metabolic disorders in the kidneys of DN. Our data further show that the linoleic acid metabolism and fatty-acids β-oxidation are significantly inhibited during DN in the db/db mouse model, suggesting that targeting its key enzymes may be a promising avenue in the treatment of DN, especially advanced-stage DN.

## Materials and Methods

### RNASeq Analysis

Raw fastq data were downloaded from the Sequence Read Archive (SRA) database under the accession number SRP237545 (Fan et al., 2019). After removing of adaptors and low-quality reads using the fastp software (version0.20.0) (Chen et al., 2018), RNA-seq reads were aligned to the human reference sequence (UCSC hg38 assembly) using HISAT2 software (version 2.1.0) (Kim et al., 2015). Then, raw counts of genes in different samples were calculated using the feature Counts software (version 2.0.0) based on the annotation obtained from GENCODE version 32^1^ (Liao et al., 2014). The differentially expressed genes between indicated groups were analyzed by the R DESeq2 package (version 1.28.1) (Love et al., 2014). The StringTie (version 2.1.0) program was used to quantify expression levels for the transcriptome in each sample at the gene levels in Transcripts Per Kilobase Million (TPM) units (Pertea et al., 2015). The difference of transcriptome in each group was evaluated by PCA analysis. Finally, the log_2_ transformed TPM was used for the following analysis.

To identify differentially co-expressed gene modules, Weighted correlation network analysis (WGCNA) was employed to the DEGs identified previously by the R WGCNA package (version 1.69) (Langfelder and Horvath, 2008). The Kyoto encyclopedia of genes and genomes (KEGG) enrichment analysis was performed for indicated modules using the R clusterprofiler package (version 3.16.1) (Yu et al., 2012). The genes shared by different pathways were also analyzed by the R clusterprofiler package (version 3.16.1) (Yu et al., 2012).

### Animals

The 8 weeks old male BKS-db/db and db/m mice were purchased from Nanjing Biomedical Research Institute of Nanjing University (License Number: SCXK 2018-008). Twelve weeks old or eighteen week mouse of db/db and db/m groups was executed to collect kidney tissues for metabolomics analyses, and 18 weeks mouse of db/db and db/m groups was executed to collect kidney tissues for proteomics analyses. For proteomics analyses, the kidneys from mice of each group were pooled together for further analysis (18 weeks db/db, *n* = 3 kidney pools, 5 mice per pool; db/m, *n* = 3 pools, 5 per pool). For metabolomics analysis, the kidneys obtained from 12 or 18 weeks mice of db/db or db/m groups were subjected to metabolomics analysis (12 weeks db/db, *n* = 4; 18 weeks db/db, *n* = 5; db/m, *n* = 8). Blood glucose levels were measured using an automated glucose monitor (Bayer, Germany). Serum creatinine was detected by using Creatinine assay kit (Jincheng, China) according to instructions. Urea assay kit (Jincheng, China) was used to quantify levels of urea nitrogen and urine protein.

### Proteomics Analysis

#### Protein Extraction

Frozen kidney tissues were added pre-cooled saline to wash away the blood. Then the tissues were grinded with liquid nitrogen and continue added Roche’s lysate (about 7 times of the weigh, μL), passed through ultrasonic break. Finally, the mixture was centrifuged at 15,000*g* for 15 min, and the supernatant was taken for the measurement of the protein concentration.

#### Enzymatic Hydrolysis

Acetone (about 4 times volume) was added to the supernatant for the protein precipitation. And the protein was re-dissolved by 8 M of urea. Trichloroethyl phosphate (TCEP) and chloroacetamide were used for opening the disulfide bonds and protecting the thiol, respectively, before protein enzymolysis. Finally, trypsin was added to protein, according to 50:1, and enzymatically react for 16 h to obtain the peptides.

#### Isobaric Labeling

iTRAQ was used as the labeling regents for two groups of peptides (db/db and db/m), respectively. After isobaric labeling, two groups of peptides were passed through pre-fractionation by High-performance liquid chromatography (HPLC). And the eluent were collected per-minute and merged into 10 fractions. Then dry the samples by vacuum centrifugation for LC-MS analysis. An Agilent 1290 UPLC with 4.6 mm × 250 mm Reprosil C18 column was used for pre-separation. The solvents were ACN (solution A) and water with NH_4_OH (pH = 10, solution B). The HPLC gradient was 0 min 5% solution A to 60 min 90% solution A with 1 mL/min of flow rate. LC-MS analysis method: an AB Nano-LC 400 (AB SCIEX, America) with a 150 μm × 100 mm AquaC18 column was used for peptides separation and an AB triple TOF 5600 mass spectrometer (AB SCIEX, America) was used for peptides analysis. The solvents were ACN (solution A) and water with 0.1% of acetic acid (solution B). The HPLC gradient was 0 min 5% solution A to 90 min 80% solution A with 1,000 nL/min of flow rate.

#### Bioinformatic Analysis

Raw LC-MS/MS files were searched against the UniProt mouse proteome database using MaxQuant software (version 1.6.17.0) enabled with Andromeda search engine (Tyanova et al., 2016). The protease was Trypsin/P. Up to two missed cleavages were allowed. Carbamidomethyl (C) was considered as a fixed modification. Variable modifications were oxidation (M) and N-terminal acetylation. The cutoff of the false discovery rate (FDR) was set as 0.01 for both proteins and peptides. The Student’s *t-*test was used to evaluate whether proteins were differentially expressed between indicated groups. Gene set enrichment analysis was conducted using the R cluster profiler (version 3.16.1) package (Yu et al., 2012). Protein–protein functional networks were constructed using the string database with default settings, and visualized by the Cytoscape (version 3.8.1) software (Shannon et al., 2003).

### Metabolomics Analysis

#### Sample Preparation

Twenty milligrams of tissue sample was crushed, and then 200 μL of ice methanol was added for the protein precipitation. Then the solution was centrifuged at 12,000 rpm for 10 min after standing at -20°C for10 min. After that, the supernatant was added 5 μL of Fmoc-Gly-OH (0.4 mg/mL) as the internal standard and analyzed by LC-MS. LC experiment was performed on Waters2695e UPLC (Waters, America) with the Fortis C18 column (2.1 × 100 mm, 1.7 μm). The column temperature was set as 30°C. The mobile phase was water containing acetic acid (99.9/0.1, v/v, solvent A) and Methanol (solvent B). A gradient of 0 min, 25% (B); 9 min, 85% (B); 13 min, 85% (B); 22 min, 95% (B); 25 min, 95% (B) was used for positive mode. A gradient of 0 min, 20% (B); 8 min, 95% (B); 12 min, 95% (B); 13 min, 98% (B); 21 min, 98% (B) was used for negative mode. The posting time was 11 min. The flow rate of the mobile phase was set at 0.3 mL/min. The injection volume was 5 μL. Mass spectrometric experiments were performed on Q-Exactive mass spectrometer (Thermo Fisher Scientific, Untied States). The detection was performed under electrospray ionization (ESI) mode with both positive and negative full scan mode. Solutions were infused from the ESI source at 0.3 mL/min with parameters: capillary 4,000 V, drying gas 12 L/min, drying gas temperature 350°C. Nitrogen was used as the nebulizing and drying gas. All MS conditions were optimized to achieve maximal detection sensitivity. Quality control (QC) samples were also prepared and collected from all the tissues. These QC samples were injected six times before the sample analysis to check the stability of the system and injected one time every six samples in sample analysis.

Thermo Data Analysis software was used for the deconvolution of the LC-MS spectrum, including baseline correction, processing noise, and peak alignment et al. Then the managed data was leaded in Compound Discoverer TM2.0 for the normalization to achieve the matrix formed by retention time, m/z, and intensity. After that, this matrix was experienced the principal component analysis (PCA) and orthogonal PLS-DA (OPLS-DA) to be obtained the differential metabolites between the groups by the R ropls package (version 1.21.0) (Thevenot et al., 2015). Metabolites with variable importance in the projection (VIP) over 1 were further compared by one-way analysis of variance (One-way ANOVA) and log2 fold change analysis, metabolites with fold change > 2 or < 0.5 and *P* < 0.05 were considered as statistically significant. Last, the differential metabolites were traced to the metabolite pathway through KEGG by MetaboAnalyst^2^ (Chong et al., 2018).

## Results

### RNASeq Data Reveals Different Transcriptome Pattern of Kidney Tissues With DN

**FIGURE 1:**
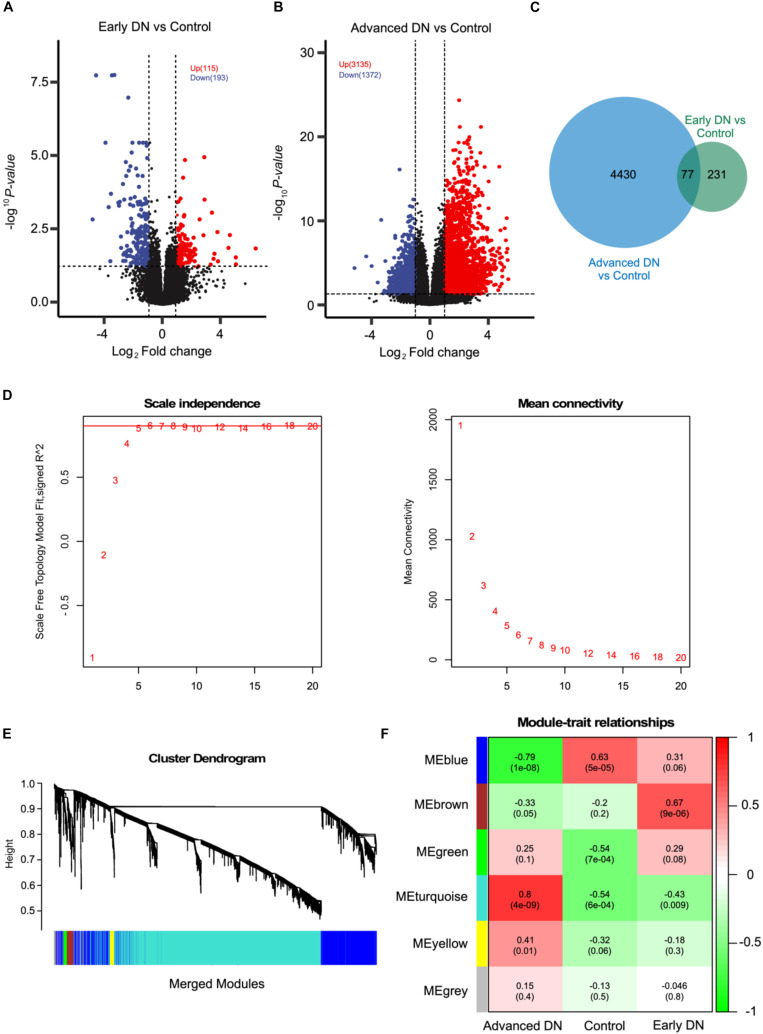
WGCNA analysis based on RNASeq data revealed six co-expression modules. **(A,B)** Volcano plot showing the DEGs between early stage DN and control **(A)** and late-stage DN and control **(B)**. **(C)** Venn diagram showing the number of DEGs commonly and uniquely expressed between indicted groups. **(D)** The scale-free fit index (left) and mean connectivity (right) of the network topology at different soft-thresholding powers. **(E)** The cluster dendrogram of DEGs of patients with DN. Each line represents one gene, and each color below represents one co-expression gene module. **(F)** Correlation analysis between different co-expression gene modules and DN phenotypes.

To investigate the molecular mechanisms during DN pathogenesis, RNASeq data of patients with early stage DN, advanced-stage DN, and non-tumor adjacent normal kidney tissues were obtained from the publicly available database. As expected, the transcriptome obviously segregated advanced-stage DN samples and other samples (Supplementary Figure S1A), suggesting there existing dramatically molecular change during DN pathogenesis. Intriguingly, the transcriptome of kidney biopsy does not change significantly in early stage DN patients compared with that of non-diabetic kidney biopsy. Next, we further conducted the DEGs analysis between kidney transcriptome of patients with early stage DN and control patients without T2D. As the volcano plot shows, 115 up-regulated and 193 down-regulated genes were identified (Figure 1A, DESeq2, *P* < 0.05 and fold change > 2 or < 0.5) (complete DEGs list can be found in Supplementary Table S1). Of these, some chemokines and cytokines such as CXCL12, IL-6, CXCL8, CCL20, CXCL1, and IL1B were significantly suppressed during early DN pathogenesis. At the same time, a lot of non-coding RNAs including MIR29A, MIR4521, PABPC5-AS1, and AL353600.2 were highly upregulated in early DN. However, the detailed function of these RNAs in DN remained further investigation.

Relative to mild transcriptome change between early stage DN and control, thousands of genes were dramatically altered in advanced-stage DN. As a result, 3,135 up-regulated and 1,372 down-regulated genes were identified using the same threshold (Figure 1B; complete DEGs list can be found in Supplementary Table S2). Interestingly, some inflammation regulators including IL-6, CXCL3, IL-6, CXCL8, CCL5, and CCL2 are significantly increased in late-stage DN, which suggests that there exists an inflammatory microenvironment in kidneys from patients with advanced-stage. Furthermore, CYP26B1, CYP4A22, COX5BP1, and other metabolism-related genes were significantly inhibited in advanced-stage DN. Together, the above results are in a line with the high level of inflammation, disrupted metabolic behavior, and tubular dysfunction observed in kidneys of patients with advanced-stage DN.

### WGCNA Reveals Detailed Molecular Dysregulations in Advanced-Stage DN Kidneys

To further characterize the molecular mechanisms of DN, we combined all DEGs identified in both early stage DN and advanced-stage DN groups. A list of 4,738 genes was found to be potentially associated with DN (Figures 1C,D). Next, we performed WGCNA analysis to explore the potential mechanisms of DN pathogenesis using above 4,738 genes. The sample hierarchical clustering results indicated there remains a large difference between these three groups (Supplementary Figure S1C). The soft-threshold analysis revealed that the accuracy of the model reached highest when the power was set as 6 (Figure 1D). Six gene co-expression modules were constructed based on WGCNA analysis (Figures 1E,F). Of these, the turquoise module containing 3,143 genes was most closely correlated with the advanced-stage DN (*R* = 0.80, *P* < 0.001). The blue module containing 1,282 genes was most closely correlated with the control group (*R* = 0.63, *P* < 0.001), while the brown module that containing 104 genes was most closely correlated with the early stage DN (*R* = 0.67, *P* < 0.001).

**FIGURE 2:**
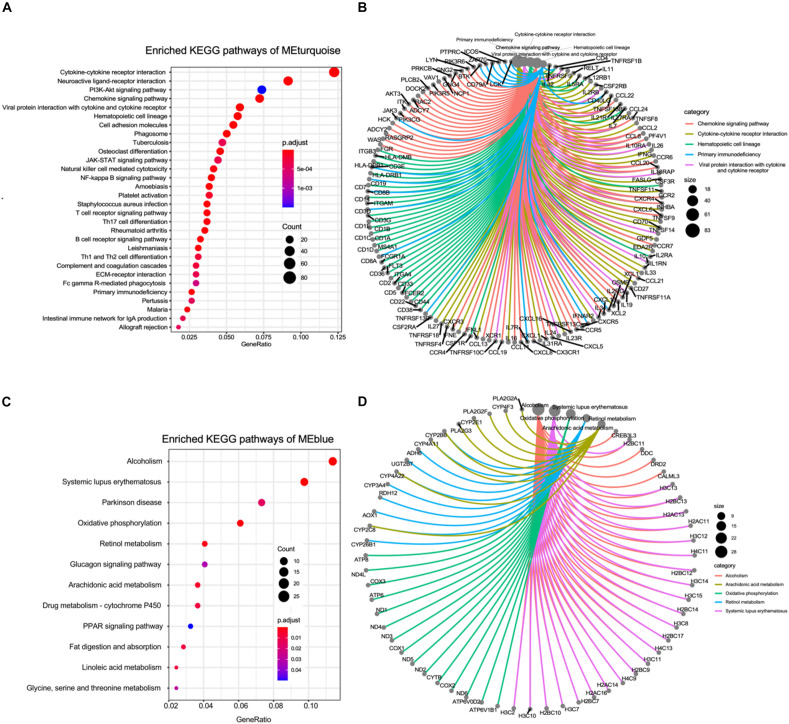
KEGG pathway enrichment of the co-expression gene modules. **(A)** The bubble plot showing the enriched KEGG pathways of genes in turquoise module. **(B)** The circle network plot showing the shared genes between several KEGG pathways enriched in turquoise module. **(C)** The bubble plot showing the enriched KEGG pathways of genes in blue module. **(D)** The circle network plot showing the shared genes between several KEGG pathways enriched in blue module.

We further performed the KEGG functional enrichment analysis to investigate modules that most closely correlated with the advanced-stage DN. As the result depicted, genes in the turquoise module were largely involved in pathways related to inflammatory regulating pathways such as Cytokine–cytokine receptor interaction, JAK-STAT signaling pathway, and T cell receptor signaling pathway. Furthermore, these genes were also found to be enriched in extracellular matrix (ECM) construction, which plays important role in kidney tubular (Figure 2A). Pathway network analysis between genes and pathways indicated that many genes related to inflammation regulation were also enriched for other pathways such as Hematopoietic cell lineage, Th1, and Th2 cell differentiation, and Primary immunodeficiency, suggesting that these genes may be associated with various biological pathways orchestrating DN pathogenesis (Figure 2B).

Next, we also conducted the KEGG functional enrichment analysis to investigate the function of genes in the blue module that most negatively (*R* = −0.79, *P* < 0.001) correlated with the advanced-stage DN. The results displayed that genes in the blue module were mainly involved in pathways related to cell metabolism pathways such as Retinol metabolism, Glucagon signaling pathway, Glycine, serine and threonine metabolism, and Linoleic acid metabolism. Additionally, these genes were also found to be enriched in Fat digestion and absorption, which play important roles in the kidneys (Figure 2C). Pathway network analysis between genes and pathways indicated that many genes related to lipid metabolism regulation were also enriched for other metabolisms related pathways such as Oxidative phosphorylation, and drug metabolisms (Figure 2D). In total, the WGCNA followed by KEGG analyses revealed that a high level of inflammation, metabolic abnormalities, and ECM construction dysfunction could play vital roles in the pathogenesis of advanced-stage DN.

### Proteomics Data Reveals That the Metabolic Disorders Are Associated With DN Pathogenesis *in vivo*

**FIGURE 3:**
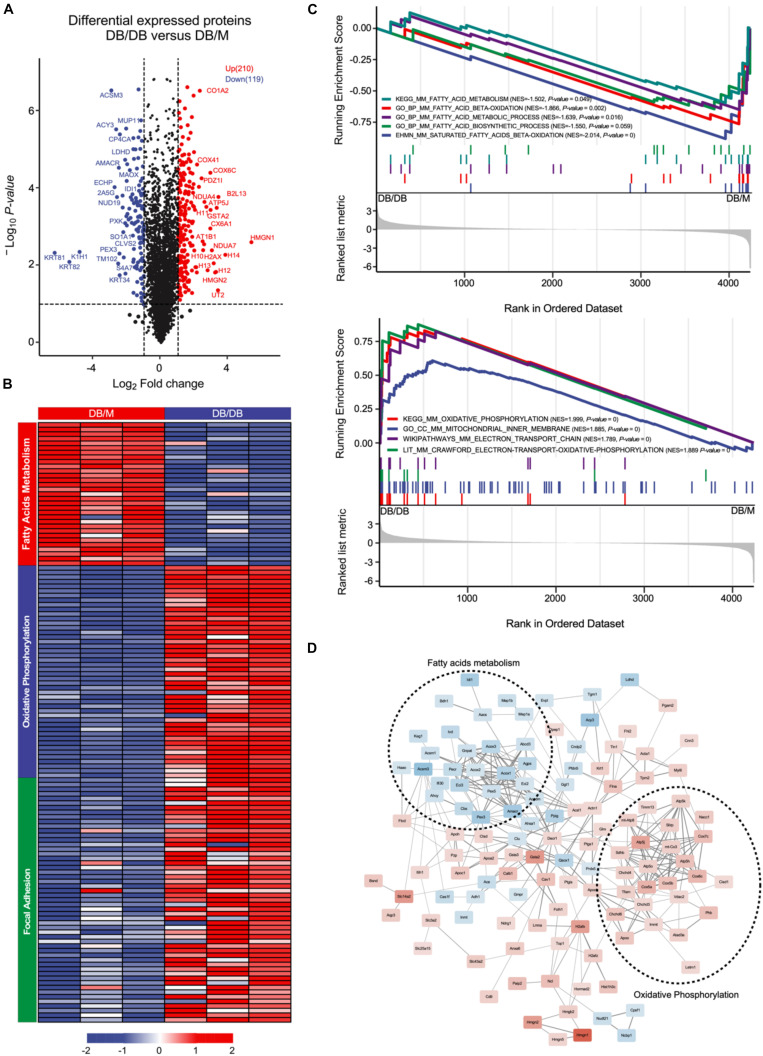
Proteomics analysis between kidneys of db/db and db/m mice. **(A)** Volcano plot showing the DEPs between kidneys of db/db and db/m mice (18 weeks db/db, *n* = 3 kidney pools, 5 mice per pool; db/m, *n* = 3 pools, 5 mice per pool). **(B)** The heatmap illustrating the expression of DEPs in kidneys of db/db and db/m mice, each row represents one protein, each column represents one sample. **(C)** GSEA plot showing the different enriched pathways in db/db (down) or db/m (up) group. **(D)** DEPs network analysis based on string database, the thickness of line represents the strength of correlation, the size of rectangle represents the fold change between db/db and db/m group, and blue means downregulated and red means upregulated.

The db/db mouse model is currently the most widely used to study the DN pathogenesis and progression (Azushima et al., 2018). To mimic the early and advanced-stage DN, the db/db mice were fed with a high protein diet for 12 or 18 weeks. After 12 or 18 weeks, the mice. The db/db mice showed significant weight gain compared with control group. Moreover, Kidney weight and kidney index were found to be significantly reduced in db/db group. The urinary protein, creatinine, and urea nitrogen levels were elevated that mimicked the DN phenotype in db/db mice (Table 1). Next, we subjected the kidneys of the db/db or control db/m mice for proteomics analysis using iTRTAQ technology. A total of 4,240 proteins was identified, and the proteome clearly segregated db/db samples and db/m samples according to PCA analysis (Supplementary Figure S1B). Next, we further conducted the DEPs analysis between db/db and db/m groups. As the volcano plot shows, 210 up-regulated and 119 down-regulated proteins were found to be significantly changed in the db/db group (Figure 3A, *P* < 0.05 and fold change > 2 or < 0.5) (complete DEPs list can be found in Supplementary Table S3). The heatmap showed that fatty-acid metabolism-related proteins including Cox2, Acox1, Acox2, Acox3, Acms1, and Acms3 were dramatically suppressed in db/db group (Figure 3B). We further performed GSEA analysis to identify pathways and biological processes level change in the db/db mice, the result revealed that fatty-acids metabolism, fatty-acids β-oxidation, fatty-acids biosynthesis pathways were significantly inhibited in db/db mice (Figure 3C), which is consistent with the analysis of human kidney biopsy transcriptome data. We further constructed the protein–protein network of the above DEPs based on the string database (Figure 3D); the network clearly displayed that plenty of enzymes involved in fatty-acids metabolism were co-inhibited in db/db mice. Therefore, our *in vivo* proteomics further confirm the important role of fatty-acids metabolism in DN pathogenesis and progression.

**TABLE 1:** Biochemical and physical characteristics of experimental groups.

Characteristic	db/m	db/db–12w	db/db–18w
	(n=15)	(n=8)	(n=8)
Body weight (g)	21.2 ± 0.6	39.6 ± 2.1**	60.6 ± 2.8**
Kidney Index (mg/kg)	14.9 ± 0.4	9.3 ± 0.3**	7.6 ± 0.3**
Blood glucose (mmol/L)	7.5 ± 1.1	15.5 ± 2.5**	18.4 ± 1.4**
Creatinine (µmol/L)	9.7 ± 3.3	14.3 ± 4.3**	23.5 ± 3.8**
Urea nitrogen (mmol/L)	10.2 ± 1.9	12.5 ± 2.4*	15.1 ± 2.9**
Microalbuminuria (µg/L)	100 ± 48.1	176.8 ± 55.9*	457.0 ± 73.5**

### Metabolomics Data Further Validates the Fundamental Role of Lipid Metabolism in DN Pathogenesis

**FIGURE 4:**
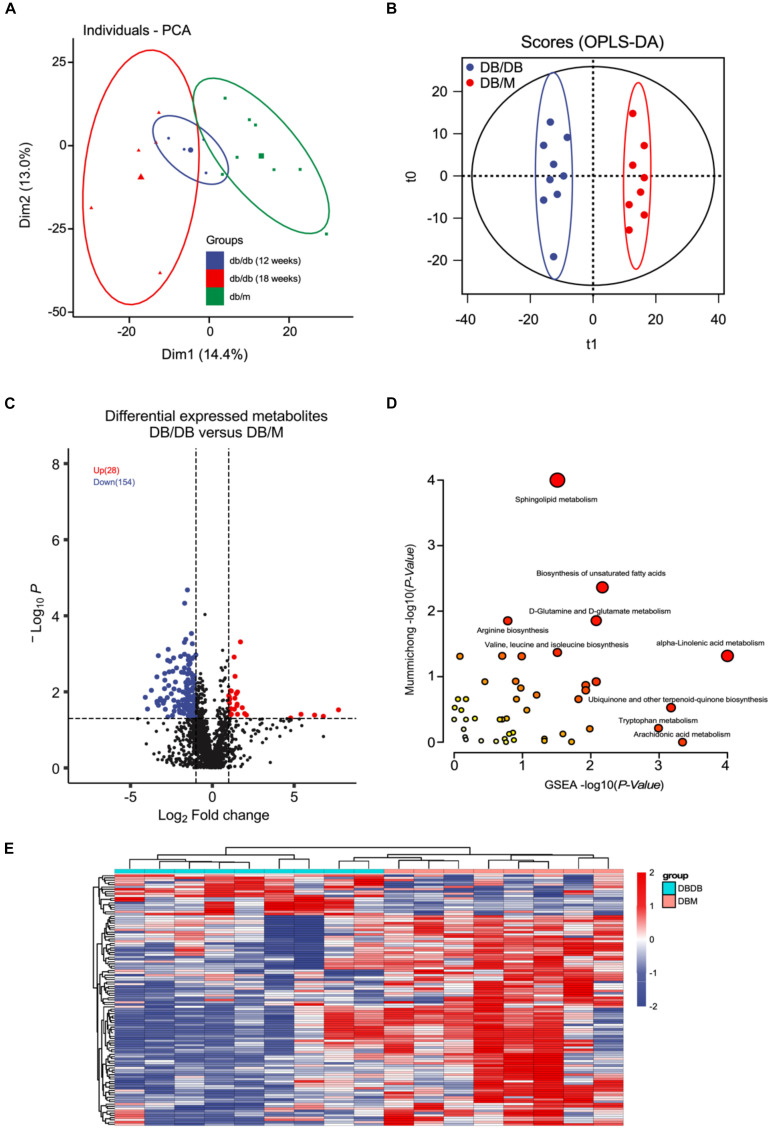
Metabolomics analysis between kidneys of db/db and db/m mice. **(A,B)** PCA **(A)** and OPLS-DA **(B)** plots of the metabolites detected in kidneys of db/db and db/m mice (12 weeks db/db, *n* = 4; 18 weeks db/db, *n* = 5; db/m, *n* = 8). **(C)** Volcano plot showing the DEMs between kidneys of db/db and db/m mice. **(D)** The bubble plot showing the enriched KEGG pathways of DEMs between kidneys of db/db and db/m mice. **(E)** The heatmap illustrating the levels of DEMs in kidneys of db/db and db/m mice, each row represents one protein, each column represents one sample.

Since the fatty-acids metabolism pathways were significantly disrupted during DN pathogenesis and progression *in vivo* as the proteomics analysis showed. We sought to performed untargeted metabolomics analysis of mice kidneys of db/db and db/m group using the LC-MS/MS method (Supplementary Figure S1D). The PCA plot showed that there existing a dramatic difference in the metabolome of kidneys from db/db and db/m mice (Figure 4A). Further OPLS-DA modeling analysis was employed to identify metabolites with significant change (Figure 4B). As a result, 28 up-regulated and 154 down-regulated metabolites were found to be significantly altered in db/db group (Figures 4C,E, *P* < 0.05 and fold change > 2 or < 0.5 and VIP > 1) (complete DEMs list can be found in Supplementary Table S4). Of these metabolites, a lot of fatty acids and their related metabolites (±)10(11)-EpDPA, (±)9-HpODE, (±)13-HODE, Linoleic Acid, and Docosahexaenoic acid were significantly down-regulated in db/db mouse kidneys. Next, we conducted metabolite enrichment analysis using the online MetaboAnalyst tool, the results indicated also that Sphingolipid metabolism, Biosynthesis of unsaturated fatty acids, and alpha-Linolenic acid metabolism pathways were involved in the pathogenesis and progression of DN.

**FIGURE 5:**
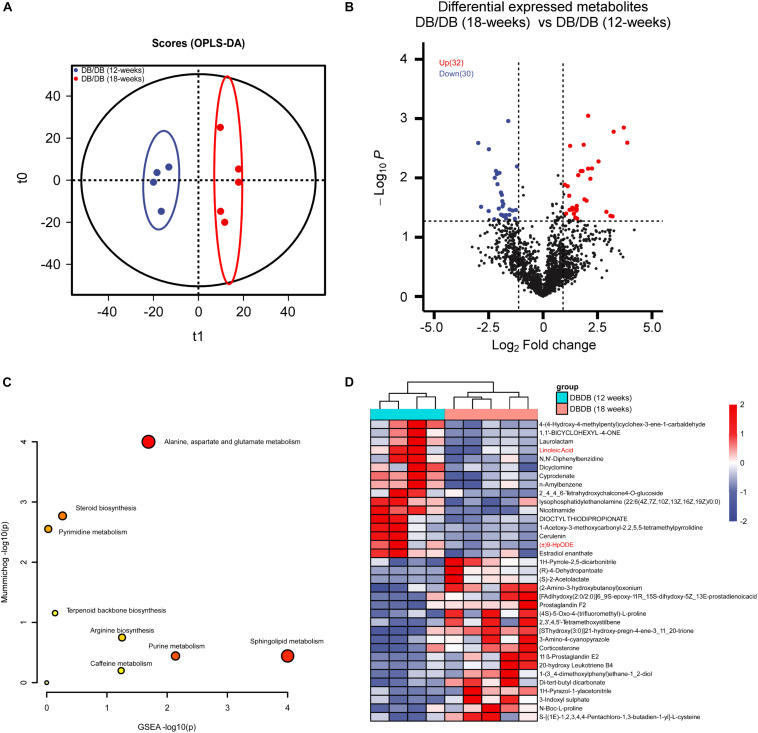
Metabolomics analysis between kidneys of early stage DN and advanced-stage DN mice. **(A)** OPLS-DA plots of the metabolites detected in kidneys of early stage DN and advanced-stage DN mice (12 weeks db/db, *n* = 4; 18 weeks db/db, *n* = 5). **(B)** Volcano plot showing the DEMs between kidneys of early stage DN and advanced-stage DN mice. **(C)** The bubble plot showing the enriched KEGG pathways of DEMs between kidneys of early stage DN and advanced-stage DN mice. **(D)** The heatmap illustrating the levels of DEMs in kidneys of early stage DN and advanced-stage DN mice, each row represents one protein, each column represents one sample.

Next, we analyzed the metabolic change of mice kidneys during DN progression. The OPLS-DA modeling analysis was employed to identify metabolites with significant change (Figure 5A). As a result, 32 up-regulated and 30 down-regulated metabolites were found to be significantly altered in advanced-stage group compared with early stage group (Figures 4D, 5B, *P* < 0.05 and fold change > 2 or < 0.5 and VIP > 1). Interestingly, (±)9-HpODE and Linoleic Acid were also found to significantly decreased during DN progression. Further metabolite enrichment analysis using the online MetaboAnalyst tool showed that Sphingolipid metabolism remained altered. Together, the *in vivo* metabolomics data also demonstrate that lipid metabolism may play important roles in DN pathogenesis and progression.

### Integrating Multi-Omics Data Indicates Targeting Fatty-Acids Metabolism Pathways May Bring Benefits for DN Patients

**FIGURE 6:**
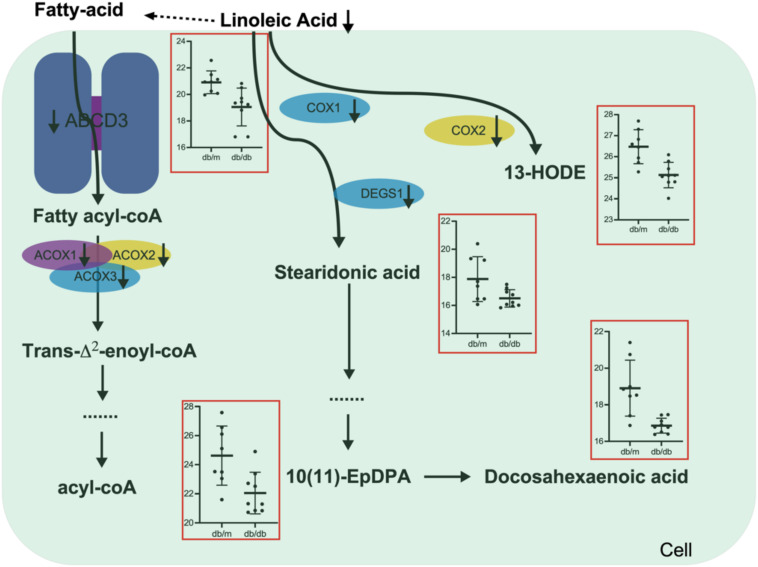
Schematic diagram of the linoleic acid metabolism and fatty-acids β-oxidation alterations during DN pathogenesis and progression.

To further confirm the role of fatty-acids metabolism pathways in DN, we treated the HK2 cells (Renal tubular epithelial cells) with high concentration glucose, the MTT results showed that high concentration glucose treatment significantly promoted cell proliferation, fibrosis and inhibited ACOX1 expression (Supplementary Figure S2). And overexpression of ACOX1 significantly rescued the phenotype triggered by high concentration glucose treatment (Supplementary Figure S2). Transcriptome, metabolome, as well as proteome data together emphasize the great importance of fatty-acids metabolism in DN. We systematically analyzed the linoleic acid metabolism and fatty-acids β-oxidation changes in DN pathogenesis (Figure 6). For the fatty-acids β-oxidation pathway, the transporter protein ABCD3 was significantly suppressed in the kidneys of db/db mice, which largely hindered the transport of long-chain fatty acids from outside to cell. Additionally, downregulation of fatty acyl-CoA activated enzymes ACOX1, ACOX2, and ACOX3 further prohibited the fatty-acids β-oxidation pathway, which in turn inhibited the kidney function of db/db mice. For the linoleic acid metabolism pathway, the linoleic acid was significantly downregulated that reduced the supply of fatty-acids β-oxidation pathway. Moreover, several key regulatory enzymes including COX1, COX2, and DES1 were simultaneously decreased, which led to the reduced level of related metabolites including 13′-HODE, stearidonic acid, docosahexaenoic acid, (±)10(11)-EpDPA in kidneys of db/db mice. The above findings shed light on that lipid metabolism, especially fatty-acids β-oxidation and linoleic acid metabolism may be potential therapeutic targets for DN.

## Discussion

DN is one of the worst T2D complications, which develops in over 40% of T2D patients. It seriously deteriorates patients’ kidney function, and eventually leads to ESRD (Fineberg et al., 2013; Thomas et al., 2015). Growing evidence suggests that the disrupted metabolism in patients with DN, as well as some *in vivo* and *in vitro* models (Kalim and Rhee, 2017). Some studies also show that supply some lipids such as ketone bodies within an optimal range may help maintain the energy homeostasis of kidneys of patients diagnosed with DN, and is likely to be beneficial for health (Tomita et al., 2020). The present study demonstrates that lipid metabolism is significantly disrupted during DN pathogenesis and progression via a combination of multi-omics data including transcriptome, proteome, and metabolome using kidney biopsies and DN mice models.

The past few decades have witnessed the great improvements of the next-generation sequencing (NGS) and LC-MS/MS technologies, which largely promote genomics, transcriptomics, epigenomics, and proteomics researches (Yang et al., 2015; Hasin et al., 2017). These technologies are widely used in the identifications of new biomarkers and drug targets and investigations of biological mechanisms of many diseases, including cancer, T2D, kidney diseases, heart diseases, and so on (Yang et al., 2015; Rinschen and Saez-Rodriguez, 2020). NGS and LC-MS/MS technologies have also accelerated the research for DN, previous studies have unveiled the molecular processes that initiate DN, influence disease progression and, mediate cell-type-specific responses to treatment (Van et al., 2017; Eddy et al., 2020). Meanwhile, cell-level biomarkers and drug targets can also be identified to assess cellular responses to external stimuli. Therefore, the researchers will gain a comprehensive molecular understanding of DN by bridging information across multi-omics data, which guides the development of targeted therapies for precision medicine.

Although we characterize the molecular change of DN at multi-omics levels, and identify the linoleic acid metabolism and fatty-acids β-oxidation as potential driven events for DN. However, the *in vivo* and *in vitro* functional assays are still needed for screening which enzyme plays the most important role in DN pathogenesis and progression, and supplying with some lipids such as ketone bodies in a reasonable range could remit glomerular sclerosis and tubular interstitial fibrosis. Moreover, artificial activation of some key enzymes in lipid metabolism should be also performed to investigate its roles in DN.

In conclusion, we systematically analyze the landscape change at RNA, protein, and metabolite levels during DN pathogenesis and progression. The molecular changes of DN contain extracellular matrix accumulation, abnormal activated inflamed microenvironment, and metabolic disorders, bringing about glomerular sclerosis and tubular interstitial fibrosis. Although lipid metabolism is found to be disrupted in DN, the comprehensive alterations at RNA, protein, and metabolite level remains unclear. Our data show that the linoleic acid metabolism and fatty-acids β-oxidation are significantly inhibited during DN in db/db mouse model, here suggest that targeting its key enzymes may be a promising avenue in the treatment of DN, especially advanced-stage DN.

## Data Availability Statement

The datasets used for this study can be found in the Gene Expression Omnibus (GEO) under accession number GSE142025.

## Ethics Statement

The studies involving human participants were reviewed and approved by the Xuzhou Medical University. The patients/participants provided their written informed consent to participate in this study. The animal study was reviewed and approved by Xuzhou Medical University.

## Author Contributions

QSh, JL, MZ, and MG performed the experiments and participated in manuscript writing. QSh and QSu designed and performed the bioinformatic data analyses. MG and QSu wrote the manuscript. HL participated in data analysis and manuscript writing. All authors contributed to the article and approved the submitted version.

## Conflict of Interest

The authors declare that the research was conducted in the absence of any commercial or financial relationships that could be construed as a potential conflict of interest.

## Footnotes

1 https://www.gencodegenes.org

2 https://www.metaboanalyst.ca/MetaboAnalyst/upload/EnrichUploadView.xhtml
