# Supplementary material for: Multi-Omics Analysis of Diabetic Nephropathy Reveals Potential New Mechanisms and Drug Targets
Source: Front Genet. 2020 Dec 11;11:616435. doi: 10.3389/fgene.2020.616435 (PMC7759603; doi:10.3389/fgene.2020.616435)
Supplement: Supplementary file 2 [file Data_Sheet_2.docx]

**Supplemental materials and methods**

**Cell culture and treatment**

Human proximal tubule epithelial cell line (HK-2, source: male) used in this study were purchased from AJCC. Cells were initially cultured in low-dose glucose DMEM medium (Gibco, USA) supplemented with 10% fetal bovine serum (FBS) (Gibco, USA) at 37 ^o^C with 5% CO_2_.

Cell density was adjusted to around 1×10^5^ cells/mL with low glucose DMEM medium and mixed by gently blowing. Then, 2 mL cell suspension was added to each well. After culturing for 12 hours, cells were cultured with high glucose (36 mmol/L), low glucose (5.56 mmol/L), and mannitol (5.56 mmol/L glucose and 30.44 mmol/L mannitol) DMEM medium containing 1% FBS for 96 hours until cellular extracts were measured.

**Plasmids and transfection**

The genomic DNA of HK-2 was isolated and the full-length of ACOX1 was amplified; the PCR products were cloned into the pcDNA3.1 expression vector (Invitrogen, CA, USA). The plasmid in the experiment was transfected with Lipofectamine 2000 reagent (Invitrogen, CA, USA) following the manufacturer’s instructions.

**MTT assay**

3-(4,5-Dimethylthiazol-2-yl)-2,5-diphenyl tetrazoliumbromide (MTT) assay was used to evaluate cell proliferation. Briefly, cells were seeded in 96-well plates at 1 × 10^4^ cells per well. Then, cells were grouped and treated with indicated treatment. 24 hours after treatment, the medium was removed and 20 μL of MTT dye solution (5 mg/mL in PBS buffer, Sigma) was added per well and incubated at 37 °C for 4 hours. Then supernatant was decanted and replaced with 150 μL DMSO. After 15 min incubation with gentle shaking at 37 °C, the absorbance was measured at a wavelength of 490 nm using microtiter plate reader.

**Western blot analysis**

Cell lysates were prepared using RIPA lysis buffer (Beyotime Bitechnology, Shanghai, China) mixed with phenylmethanesulfonyl fluoride at 4 °C for 40 min. The same amount of protein from each group was separated by 10% sodium dodecyl sulfate-polyacrylamide gel electrophoresis and transferred onto a nitrocellulose. The membrane was further blocked with 5% BSA for 2 hours at room temperature and incubated with primary antibodies overnight at 4 °C. Then, membranes were incubated with the secondary antibody (Santa Cruz, CA; 1:3000) for 1-2 hour at room temperature. The signals were visualized using ECL reagents (Millipore, USA). The specific antibody dilution ratios were as follows: anti-ACOX1 antibody (1:500, ab238939, abcam, USA) and anti-TGF-β1 antibody (1:800, ab215715, abcam, USA). β-actin was used as an internal control.

**Immunofluorescence analysis**

HK-2 cells were seeded with a density of 10^5^ cells per well and incubated at 37°C, 5% CO 2 for 48 hours. Then, the cells were fixed with 4% paraformaldehyde, permeabilized with 0.1% Triton X-100 for 5 minutes, and blocked with nonspecific antigens with 5% BSA. The cells were incubated with anti-ACOX1 antibody (1:500, ab238939, abcam, USA) and anti-TGF-β1 antibody (1:800, ab215715, abcam, USA) for 15 hours at 4°C, followed by PE-conjugated secondary antibody (Invitrogen, Carlsbad, CA) at room temperature for 1 hour, and the fluorescent nuclear DAPI stain (Invitrogen, Eugene, OR) for 5min. Then fluorescent images were observed under a confocal laser scanning microscope (Nikon, Japan).


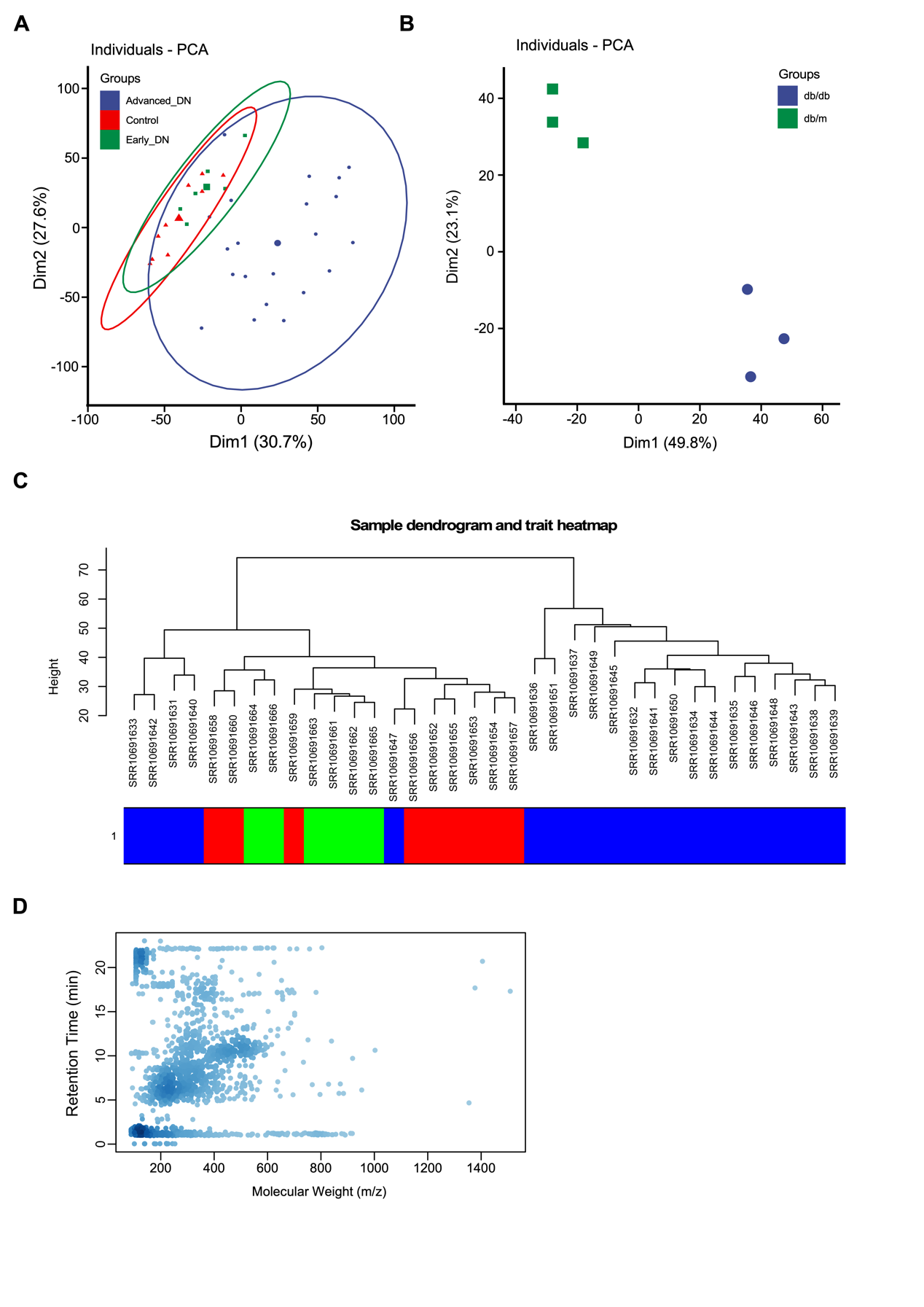


**Figure S1 (A-B)** PCA plots of different samples based on transcriptome **(A)** or proteome **(B)** data. **(C)** The cluster dendrogram of samples from patients with DN. **(D)** The scatter plot showing the relation between molecular weight and retention time of each metabolite detected in mice kidneys.


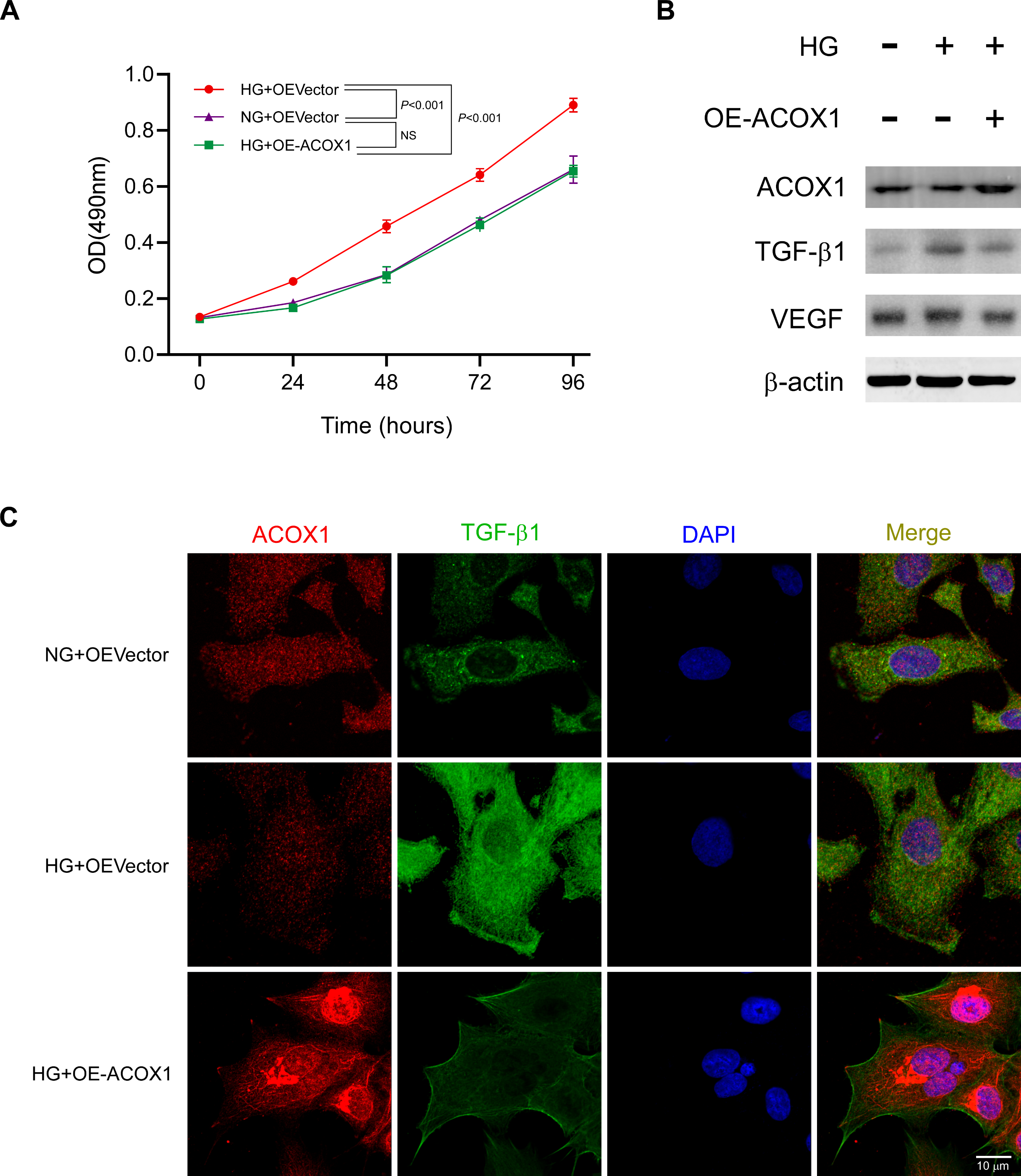


**Figure S2 (A)** MTT assay was performed to assess the proliferation ability of HK-2 cells with different treatment. Data were collected every 24 h. **(B-C)** Western blot **(B)** and immunofluorescence **(C)** analyses of indicated proteins in HK-2 cells after different treatment.
